# Comparative developmental osteology of the seahorse skeleton reveals heterochrony amongst *Hippocampus* sp. and progressive caudal fin loss

**DOI:** 10.1186/2041-9139-5-45

**Published:** 2014-12-22

**Authors:** Tamara Anne Franz-Odendaal, Dominique Adriaens

**Affiliations:** Mount Saint Vincent University, Halifax, Nova Scotia Canada; University of Ghent, Ghent, Belgium

## Abstract

**Background:**

Seahorses are well known for their highly derived head shape, prehensile tail and armoured body. They belong to the family of teleosts known as Syngnathidae, which also includes the pipefishes, pipehorses and seadragons. Very few studies have investigated the development of the skeleton of seahorses because larvae are extremely difficult to obtain in the wild and breeding in captivity is rarely successful. Here we compare the developmental osteology of *Hippocampus reidi* over an ontogenetic series spanning the first 93 days after release from the brood pouch to that of a smaller series of *Hippocampus;* namely *H. subelongatus.*

**Results:**

We compare the osteology in these two species over growth to the published description of the dwarf species, *H. zosterae*. We show that ossification onset in *H. subelongatus* is earlier than in *H. reidi*, despite similar sizes at parturition. Interestingly, the timing of development of the bony skeleton in *H. zosterae* is similar to that of the larger species, *H. subelongatus*. Furthermore, we show that the growth rate of all three species is similar up until about 30 days post pouch release. From this stage onwards in the life history, the size of the dwarf species *H. zosterae* remains relatively constant whilst the other two species continue growing with an accelerated growth phase.

**Conclusion:**

This data together with a phylogenetic assessment suggests that there has been a heterochronic shift (a delay) in the timing of ossification in *H. reidi* and accelerated bonedevelopment in *H. zosterae*. That is, *H. zosterae* is not a developmentally truncated dwarf species but rather a smaller version of its larger ancestor, “a proportioned dwarf” species. Furthermore, we show that caudal fin loss is incomplete in *Hippocampus* seahorses. This study shows that these three species of *Hippocampus* seahorses have evolved (either directly or indirectly) different osteogenic strategies over the last 20–30 million years of seahorse evolution.

## Background

**Figure 1:**
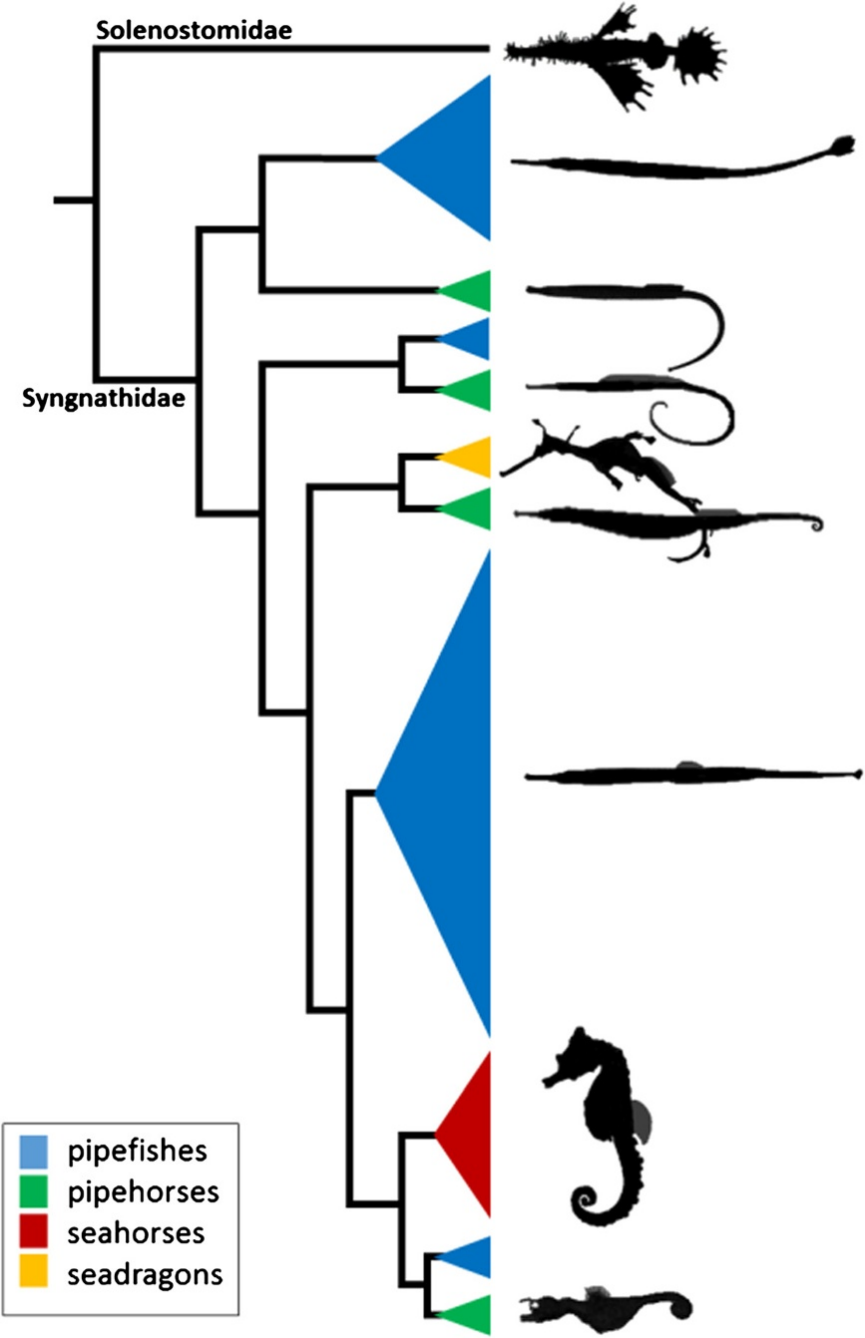
**Cladogram showing the overall relationships between pipefishes, pipehorses, seadragons and seahorses.** Note that the paraphyletic nature of ‘seadragons’ is not indicated in this figure. Cladogram is modified from Hamilton *et al.*[5].

The skeleton of the Syngnathidae, with their elongated body shape and flexible trunk and tail, has attracted the attention of skeletal biologists over the last decade. Syngnathidae includes the seahorses (*Hippocampus*), pipefishes (e.g. *Syngnathus*), pipehorses (*e.g. Syngnathoides*) and seadragons (e.g. *Phyllopteryx*) (Figure 1). This family comprises more than 230 living species [1]. Only ‘seahorses’ represent a monophyletic clade by consensus, whereas the other vernacular names reflect paraphyletic morphotypes [2–5] (Figure 1). The basal morphotype for Syngnathidae is an elongated body that is positioned horizontally in the water column when swimming (e.g. the pipefish). The well-known seahorse morphotype is similar except that seahorses position their body vertically while swimming, their head has a tilted position with respect to the body and they have a prehensile tail. Pipehorses can be very similar to pipefishes in their morphology except that they have a prehensile tail (e.g. *Syngnathoides biaculeatus*) or they can resemble the seahorse morphotype (*e.g.* pygmy pipehorse, *Idiotropiscis*). Seadragons, on the other hand, are distinguished by their elaborate extensions of the skin (*e.g.* weedy seadragon). Interestingly, the caudal fin is only observed in adult pipefishes [4, 6].

Within seahorses, *Hippocampus* has 55 recognized species [7], although several undescribed species suggest that this number may be as high as 80 species [8]. Species range in size from one centimeter to about 30 cm, and exhibit variation in their overall morphology, such as variation in snout length [9], colour [8] and integumentary structures [6]. The latter can make them highly cryptic (*e.g. H. bargibanti* mimicking the soft corals they live amongst) [8]. The habitat preferences of seahorses can also be quite specific (e.g. sea grasses, algal or spongy coral reefs); the evolutionary origin of seahorses has been linked to such niches (sea grass beds) becoming available [2].

Studies pre-2005 have described the overall seahorse body shape from juvenile stages through to adults (e.g. [10–12]; and others cited in [13]), while other more recent publications only describe a handful of juvenile stages (e.g. [14]). The allometric growth in several seahorses (e.g. *Hippocampus kuda*) has also been described with specific focus on their derived skull morphology and snout elongation (e.g. [9, 13, 14]) or on their suction feeding kinematics (e.g. [15–17]). Few studies, however, describe the timing of the development of the skeleton in seahorses over growth. The main reason for this is that obtaining embryonic seahorses is practically impossible and obtaining specimens while still in the pouch is limited and only possible if adult males are sacrificed. Similarly, obtaining young seahorses, soon after pouch release, is extremely challenging and requires a gravid male.

Of the handful of studies that describe the seahorse skeleton in some detail, all use an acid based staining procedure that is now known to decalcify small bones [18–20]. The detailed study by Azzarello [21] describes skeletal development of the dwarf seahorse, *Hippocampus zosterae*, and the pipefish, *Syngnathus scovelli* from embryonic to juvenile stages up to 51 and 53 days post hatching, respectively. A more recent study describes the skeleton of *Hippocampus reidi*[22]*.* This study, published in Portuguese, however, only describes the first 35 days after birth (i.e. pouch release); it again uses the less optimum decalcifying staining protocol for analyses. Therefore, a re-examination of post-hatching skeletal development in *Hippocampus* using an acid-free staining protocol is warranted.

Miniaturisation is a widespread phenomenon amongst vertebrates, indeed amongst metazoans, and allows dwarfed forms to partition resources and occupy ecological niches that are otherwise inaccessible or unsustainable for their larger relatives [23–27]. Indeed, minaturisation has independently evolved at least 34 times (in South American freshwater fishes alone [28]. Miniaturised animals often have a reduction or simplification of various structures and organs [29, 30]). They may simply be dwarfed but otherwise identical to a larger ancestor [31, 32] (truncated growth) or they may resemble an early developmental stage of the larger ancestor (i.e. developmentally truncated). Importantly, dwarfism may not affect all systems equally such that both proportioned and asymmetrical dwarfs can exist. Developmental studies describing miniaturised species frequently point to heterochrony as a major mechanism [24]. Heterochrony is a change in the relative timing of developmental processes so that an event occurs earlier, later or at a different rate in a taxon compared to its ancestor. However, in practice, developmental timings of ancestors are virtually never available and therefore almost all studies of heterochrony involve changes in timing amongst related taxa [33]. Paedomorphosis is usually implicated as the evolutionary pattern correlated with the reduction in size and associated morphological changes. However, the actual mechanism leading to size reduction can vary amongst taxa [34]. Together with the reduced size of miniaturised forms, the accompanying morphological changes could include a reduction or structural simplification, hyperossification (compensating for a weaker skeleton caused by simplification), morphological novelty (as a consequence of skeletal rearrangements), and increased variability in the elements that form late in development. A good example of this is *Danionella dracula*, a developmentally truncated dwarf cypriniform (about half the size of zebrafish) that exhibits hyperossification in its skeleton and which has evolved a morphological novelty (long, pointed odontoid processes in its oral jaws) [35].

Here, we compare cranial bone development of *Hippocampus reidi* (the long nose seahorse) over a large growth series (up to 95 days post birth) to that of a similarly sized but not closely related species, *Hippocampus subelongatus* (the western Australian seahorse) [36]. Considering that these two species are significantly larger than the dwarf seahorse, *H. zosterae*, we compare our data to the published osteogenetic sequence for this species [21]. Our objectives are to determine whether skeletal ontogeny in the two larger species is similar to one another and how these compare to the dwarfed seahorse. Does the dwarfed species show morphological changes typical of miniaturised or smaller forms (i.e. altered ossification sequences or onsets) compared to the larger species? To test this, we compared bone development in these three *Hippocampus* species of different body sizes (Table 1), and examined the onset/offset of ossification. Additionally, we focus on the caudal fin, which is considered in the context of its evolutionary loss in derived syngnathid taxa, such as seahorses.

**Table 1:** **Maximal size of adult seahorses of the species studied (based on data from Fishbase; TL = total length)** [7]

	Max adult size (TL)	Size at maturity (TL)
***H. reidi***	17.5 cm	8 cm
***H. subelongatus***	20-22 cm	13 cm
***H. zosterae***	5 cm	2.1 cm

## Methods

Seahorses (*Hippocampus subelongatus* syn. *angustus [Castelnau 1873], H. reidi [Ginsburg 1933], H. zosterae [Jordan and Gilbert 1882]*) were obtained from a commercial trader in ornamental fish (deJong Marine Life, the Netherlands). For *H. reidi* an ontogenetic series was obtained from spontaneous reproduction in a laboratory fish tank. Specimens were sedated and killed in an overdose of MS222 (in accordance with Belgian legislation on the use of laboratory animals), and fixed in 10% of neutral and buffered formalin at different timings after birth. As such, an ontogenetic sample was obtained that ranged in age and size from 1 dpp (as the moment of fertilization or hatching within the pouch could not be established, we use ‘days post parturition’) to 93 dpp. In total, 44 *H. reidi* specimens were examined and measured. For *H. subelongatus* a similar spontaneous reproduction was obtained in the tank, where an ontogenetic series was obtained, ranging in age and size from 1 dpp to 8 dpp (n = 20, juveniles would not feed and all died after 8 dpp). All newly born seahorses were fed ad libitum with newly hatched *Artemia* nauplii on a daily basis and were reared at 23°C for *H. subelongatus* and 26°C for *H. reidi,* both at a salinity of 31 g/kg. For *H. zosterae* the published skeletogenic study by Azzarello [21] was used for comparative purposes. All experimental procedures were performed in accordance with the Experimental Animal Ethics Committee of Ghent University, Belgium.

Fish were cleared and stained according to the acid-free double whole mount staining protocol of Franz-Odendaal [37]. Briefly, samples were bleached, stained for cartilage and bone in an acid-free double stain using alcian blue stain (for cartilage) and alizarin red S (for bone). Samples were then trypsinized to remove opaque tissues and cleared in a glycerol series. Based on this staining, skeletal elements from multiple samples were scored as follows: 0, skeletal element absent; 1, skeletal element present; 2, skeletal element starts to ossify; 3, skeletal element is well or fully ossified. Using this scoring methodology, endochondral bones receive a score of 1 if cartilage is present, a score of 2 when ossification is first present, and a score of 3 when the element is heavily ossified. Intramembranous bones, on the other hand, are scored as 2, ossification first present, or as 3, heavily or fully ossified. Regardless of mode of ossification, ossification onset is scored as 2. It is important to note that in very young fish, alcian blue can be taken up by non-cartilaginous elements. Additionally, some intramembranous elements can take up this stain indicating that they are present (even though cartilage is not present); these elements are scored as 1 simply because they are visible in whole-mount stained fish. For endochondral bones, the presence of chondrocytes was confirmed by high magnification analysis where applicable.

The maximum length of the head (HL), tail (TL) and trunk (TrL) was measured as described by Lourie [38] using the Nikon NIS software and a Nikon SMZ1000 stereomicroscope. The total length (TL) of all specimens was then calculated as the sum of head length (HL), tail length (TaL) and trunk length (TrL). Although sexual dimorphism is known to exist in adult *Hippocampus* (with males having longer tails), this unlikely affected our results since our samples are juveniles that are not yet sexually mature.

### Histology

A *Hippocampus reidi* specimen at one day post parturition was fixed in a 4% buffered and neutralized formalin solution and stored in alcohol 70%. The specimen was decalcified using Decalc at 25%, dehydrated through a graded alcohol series, and embedded in Technovit 7100 (Heraeus, Kulzer). Semi- thin sections (2 µm) were cut using a Leica Polycut SM 2500 sliding microtome equipped with a wolframcarbide coated knife, stained with toluidine blue, mounted with DPX, and covered. Images were taken using a Reichert-Jung Polyvar light microscope (Reichert Depew, USA), equipped with a Colorview 8 digital camera (Olympus).

## Results

**Figure 2:**
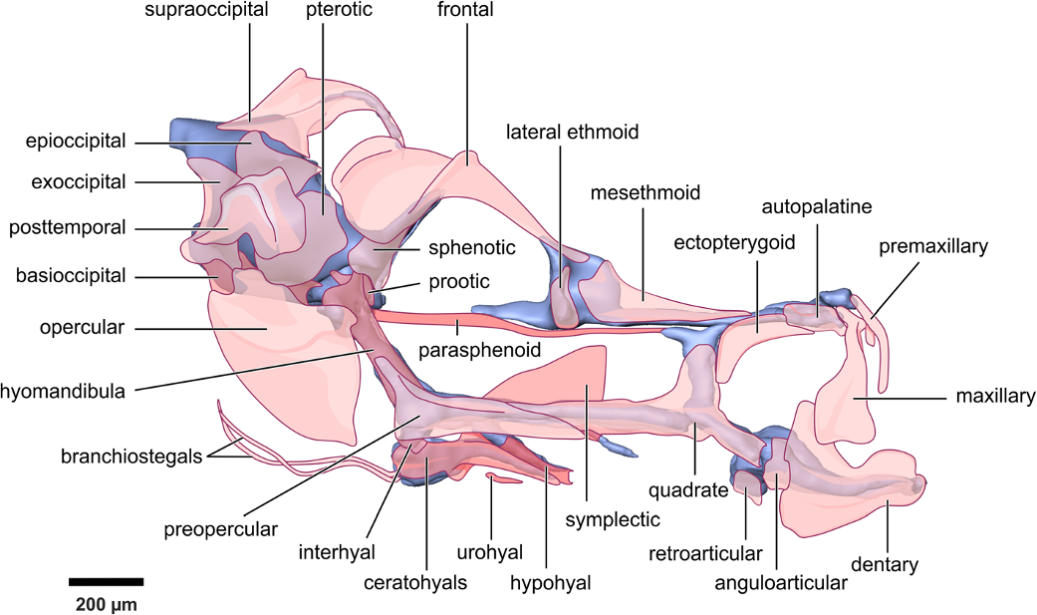
**A typical adult seahorse skull showing the skeletal elements.** Modified from [17].

**Figure 3:**
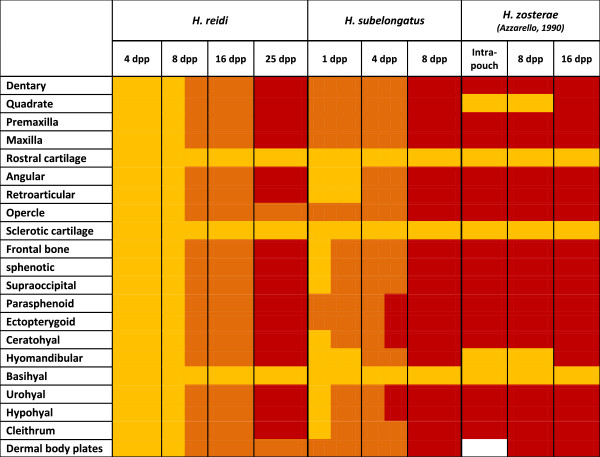
**Ossification onset in major skeletal elements in** ***Hippocampus reidi*** **,** ***H. subelongatus and H. zosterae*** **.** The scores of 0, 1, 2 and 3 are colour coded as white, yellow, orange and red, respectively. 0, skeletal element is absent (white); 1, element is present but not yet ossified (yellow); 2, element starts to ossify (orange); 3, element is well or fully ossified (red). See text for details. Data for an intra-pouch stage (5.5 mm notochordal length) is given for *H. zosterae,* this data is from Azzarello [21].

**Figure 4:**
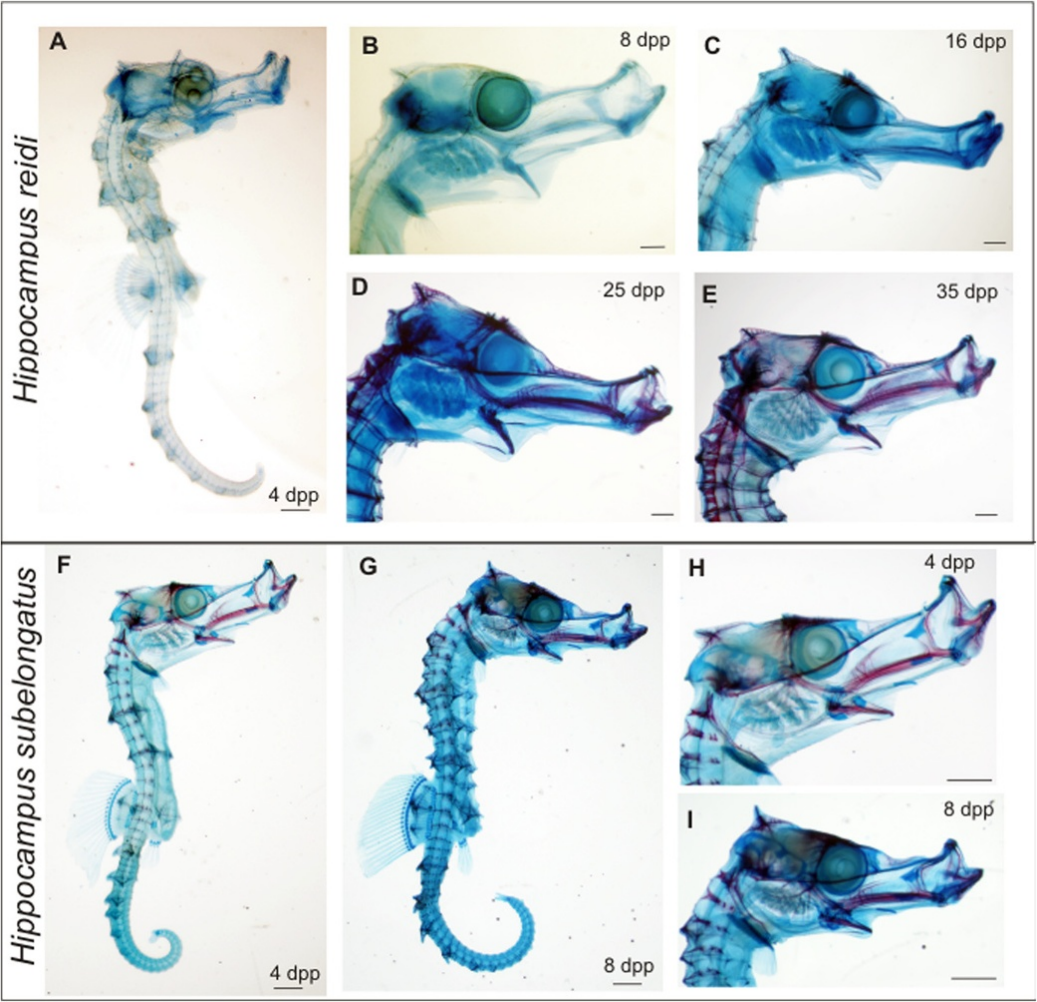
***Hippocampus reidi*** **and** ***H. subelongatus*** **examined in this study. A-E)** *H. reidi* at 4 to 35 dpp. Ossification is first visible at 16 dpp and the skull is heavily ossified by 35 dpp. **F-I)** *H. subelongatus* specimens at 4 dpp and at 8 dpp showing ossification present by 4 dpp. High magnifications of the head are also shown. Scale bars are **A)**, **B)** is 200 µm, and **C-E)** are 300 µm and in **F-I)** are 500 µm.

We assessed the timing of ossification in *H. reidi* and *H. subelongatus* and compared this to published work for *H. zosterae*, taking particular note of the onset of ossification. A typical adult seahorse skull is shown in Figure 2. Our ossification data is described below and summarised in Figures 3–4. Since substantial differences were noted, we then analysed the growth rates of these animals in order to determine whether entire suites (or regions) of bones have a shifted ossification onset/offset or alternatively whether the growth of one species is slowed or accelerated compared to another.

### Comparative analysis of bone development in *H. reidi*and *H. subelongatus*

In order to assess the osteology of these species, we analysed cleared and stained specimens in which alcian blue stains cartilaginous elements and alizarin red stains ossified elements (i.e. bone). Since Van Wassenbergh [17] already showed in a 3D reconstruction of a 1 dpp *H. reidi* specimen, that many skeletal elements are already present at this age, our assessment confirmed these findings. For example, at 4 dpp, the largest and most readily identifiable elements include the oral jaw bones and associate elements (dentary including Meckel’s cartilage, premaxilla, maxilla, quadrate, ceratohyals), the parasphenoid, the cleithrum, the coronet (a small ossicle at the level of the supraoccipital bone), the occipital bones, and the radials of the fins, to name a few (Figure 4A). At this stage the pectoral and dorsal fin radials number 16 and 17, respectively. By 8 dpp, ossification of the oral jaw elements has just started (visible as faint red staining); this ossification is more pronounced by 16 dpp and by 25 dpp ossification is evident throughout the skeleton (Figure 3, Figures 4 B, C). The skull is heavily ossified at 71 dpp (not shown) and the pectoral and dorsal fin rays number 17 and 18, respectively, at this age. Anal fin rays remain at four in number from 4 dpp to 71 dpp. No fin rays have mineralised in any of the stages examined. In summary, in *H. reidi,* chondrogenesis is well established by 1 dpp whilst osteogenesis only begins at 8 dpp.

In *Hippocampus subelongatus*, the earliest sign of ossification based on alizarin red staining is seen at 1 dpp in the oral jaws, parasphenoid, quadrate, hyoid arch bones and in a few post- and suborbital elements. By 4 dpp, several other elements have ossified and these specimens are significantly more ossified than *H. reidi* of the same age (Figure 4). By 8 dpp, most of the *H. subelongatus* skull has ossified (Figure 3, Figures 4F-I). Ossification therefore, has an earlier onset in *H. subelongatus* compared to *H. reidi*. This is interesting because *H. reidi* and *H. subelongatus* have approximately the same standard length at 4 dpp (7.5-8 mm) despite their different adult sizes (Table 1). The head (snout) length is also similar in both species at 4 dpp, although *H. subelongatus* has a deeper head at this stage. Overall very little variability was observed amongst the specimens with respect to skeletal development.

### Onset of ossification in *Hippocampus*

A comparison of the onset of ossification in *H. reidi* and *H. subelongatus* is shown in Figure 3 and we include the published data for *H. zosterae* from Azarello [21] for additional analyses. Our data clearly shows that *H. subelongatus* is at a more advanced stage of ossification four days after release from the pouch (4 dpp) than *H. reidi*, despite their similar size at parturition (±6 mm SL). It is also likely therefore that *H. subelongatus* is at a more advanced stage of development when it is released from the pouch (i.e. at birth) and that in this species some ossification occurs inside the pouch prior to parturition. In *H. zosterae*, all the elements in Figure 3 ossify prior to birth [21] and thus ossification onset is earlier than the larger species. Interestingly, the basihyal ossifies particularly late (at 65 dpp) in the sequence in *H. zosterae*; similar delays are observed in *H. reidi* and *H. subelongatus* suggesting that the late ossification of the basihyal is consistent for the entire genus. In summary, the onset of ossification in each of the three *Hippocampus* species examined here differs from one to the other. *H. zosterae* ossifies earlier compared to the two non-dwarf species and *H. subelongatus* (which is larger than *H. reidi* as an adult) has an intermediate timing of osteogenic onset compared to the other two species. There does not appear to be differences amongst these three species with respect to ossification timing within particular skeletal systems (e.g. mandibular complex, post-orbital region, etc.), and thus we conclude that there has likely been a shift in ossification for the entire suite of bones examined.

### The caudal fin

**Figure 5:**
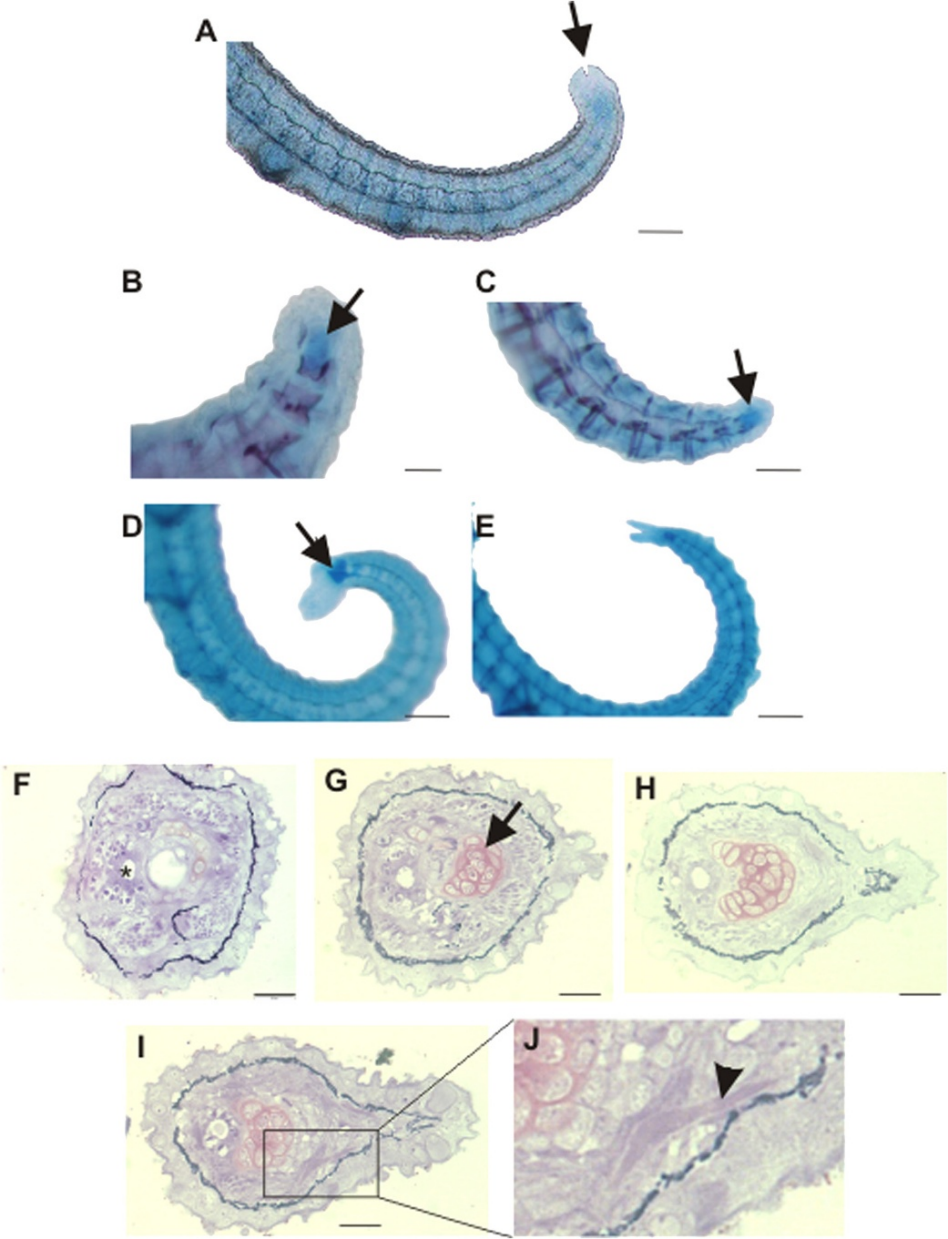
**The caudal fin in** ***Hippocampus reidi*** **and** ***H. subelongatus*** **. A-B)** whole mount staining showing the cartilage nodule (arrows) in *H. reidi* **(A-C)** and *H. subelongatus* **(D, E). A)** 5 dpp, **B)** 27 dpp, **C)** 35 dpp, **D)** 4 dpp, **E)** 8 dpp. **F-J)** transverse serial sections at the rostral border of the caudal cartilage of *H. reidi* at 1 dpp showing cartilage (arrow) extending beyond the end of the notochord (asterisk). In I-J, two hemitrichs of the caudal fin rays (arrowhead) can be seen. Histological sections are stained with toluidine blue. Scale bar in A is 100 µm and in C is 50 µm. Scale bars in B, and D-E are 150 µm, and F-I are 20 µm.

One of the most striking differences between these three *Hippocampus* species is in the caudal fin. At 4–5 dpp both *H. reidi* and *H. subelongatus* bear a small caudal fin (Figures 5 A, D). In *H. reidi*, two small elements of bone matrix could be discerned in the histological sections of the 1 dpp specimen, suggesting the presence of a primordial fin ray (Figures 3F-J). In *H. subelongatus*, two larger and one small fin ray seem to support the dorsal and ventral fin lobe, respectively (Figure 4D). Furthermore, in both species, an alcian blue stained nodule is visible at the end of the tail (Figures 5 A-E). This nodule was confirmed to be cartilage via histological staining at 1 dpp in *H. reidi* (Figures 5 E-I) and persists through to at least 71 dpp in this species, despite lack of fin rays later in development (i.e. in adults). This cartilage is likely a remnant of the caudal fin endoskeletal anlage. In *H. zosterae*, no caudal fin rays are present, however Azarello [21] does show a cartilage nodule at the tip of the tail (Figure plate 9D in [21]). In summary, all three *Hippocampus* species examined here have a cartilaginous nodule at the tip of the tail, where the larger species both have a caudal fin supported by fin rays during the early life stages. This data suggests that these species are at different stages of caudal fin reduction (see Discussion).

### Growth rates of *H. reidi*and *H. subelongatus*in comparison with that of the dwarf seahorse, *H. zosterae*

**Figure 6:**
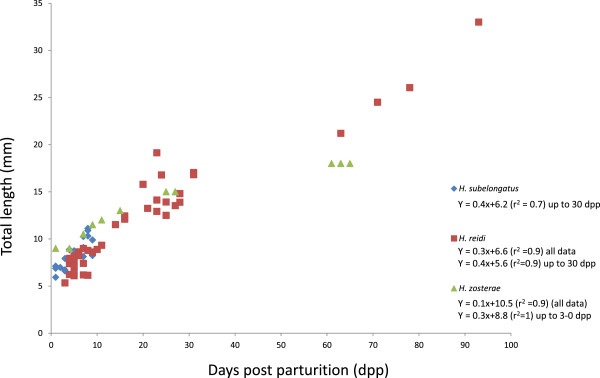
**Growth curves of** ***Hippocampus reidi*** **and** ***H. subelongatus*** **together with published growth data for** ***H. zosterae*** **from Azarello** [21]**.** Total length is given on the y-axis (mm) while the x-axis gives days post parturition (dpp). Regression equations and r-squared values for the full data set and for the period up to 30 dpp are given.

In order to compare the observed differences in ossification onset with growth, we measured the total length of the seahorses in our series. The difference in rate of growth and ossification between *H. reidi* and *H. subelongatus* is summarized in Figures 3 and 6, together with the published data on *H. zosterae*. Interestingly, all three species have a similar total length at birth (6–9 mm TL) and a similar initial growth rate up until about 30 dpp (Figure 6). At 30 dpp, all three species have a similar body length, approximately double the size at birth. After this, the growth of *H. zosterae*, the dwarf species, slows down substantially (or ‘stagnates’; slope of regression line is zero) while growth *H. reidi* (we have no data for *H. subelongatus*) continues at the same original rate until at least 90 dpp. This data demonstrates that the large size of *H. reidi* (and possibly also *H. subelongatus*) is achieved by an extended period of accelerated growth from about 30 dpp, compared to *H. zosterae*. In the dwarf seahorse, growth becomes asymptotic (probably) shortly after 30 dpp whereas *H. reidi* is still in a phase of accelerated growth. From our data, there are no indications that the larger species, *H. reidi,* goes into an asymptotic phase before 90 dpp.

## Discussion

In this study we have examined the onset of ossification and the rate of growth in three species of seahorses (genus *Hippocampus*), one of which is a dwarfed species (body size of 5 cm SL) and the other two being larger species with a body size greater than 15 cm SL. Our data shows that the two larger seahorses examined (*H. reidi* and *H. subelongatus*) demonstrate a dramatically different timing of ossification to one another with *H. subelongatus* having an earlier onset of ossification compared to *H. reidi*. That is, *H. reidi* may be delayed compared to *H. subelongatus* with respect to ossification. Alternatively, ossification in *H. subelongatus* is accelerated compared to that in *H. reidi*. Interestingly, the dwarf species, *H. zosterae,* is at an advanced stage of bone development at parturition compared to both the other two species. Furthermore, by analysing the body sizes of the seahorses in our series, we show that all three species have a similar growth rate up until about 30 dpp after which the dwarf species appears to stop growing.

Heterochrony involves a shift in the timing of developmental processes, such that an event occurs at a different rate or at a different start or offset time in one taxon compared to its ancestor [31, 39]. Since the timing of developmental events in ancestors is virtually never known, studies of heterochrony usually involve changes in timing among related taxa. Importantly, the direction of the heterochronic shift is not the same in every vertebrate clade. Our data in *Hippocampus* shows a heterochronic shift with respect to osteogenic timing when comparing these three species. Developmental heterochronies relating to the skeleton have been linked to dramatic differences in life histories of related species, for example, in altricial versus precocious species (e.g. in [40, 41]). Weisenbecker and colleagues found heterochronic shifts in development when comparing ossification of the hind limbs of marsupials to placental mammals and explained these results by the active movement required by altricial neonate marsupials [41]. In the Atlantic char, a teleost, the onset of ossification occurs earlier in dwarf morphs of this species [42]. Therefore, the shifts in ossification timing observed in these *Hippocampus* species are not uncommon amongst vertebrates.

Potentially confounding our analyses is the temporal period within the pouch, since this is not known for these species. It is possible that each of the three species has different brood times. For *Hippocampus abdominalis*, a large seahorse reaching 35 cm in maximum length (average is 18 cm SL;[7]), Woods [43] reported brooding time by males to be 34 ± 3.42 days (mean ± 1 SE) and to be highly dependent on water temperature. In this species, the mean body size at release from the pouch was 15.67 ± 0.499 mm (mean ± 1 SE), which is substantially larger than the three species we examine here (±5-7 mm SL). Data on brood times for other *Hippocampus* species could not be found.

Phylogenetic analyses of *Hippocampus* yields different hypotheses for the evolutionary relationships amongst these three species (e.g. [36, 44]). Using a more comprehensive dataset of nuclear markers, Teske [36] indicated that *H. zosterae* and *H. reidi* are more closely related to one another than either is to *H. subelongatus*. This would suggest that the ossification timing in *H. subelongatus* is the basal condition for the seahorses studied here (the actual ancestral ossification timing of these three species is not known, of course). Linking the evolutionary relationships to our observations, the most parsimonious hypothesis with respect to ossification timing is that *H. zosterae* displays a heterochronic shift in ossification to earlier in development while *H. reidi* displays the opposite shift by delaying ossification. Clearly, there must have been some selection pressure for ossification to shift in either direction, either acting directly on the organism, or on the gene networks. Alternatively, there could be a secondary (indirect) effect of selection acting elsewhere. It is typical to observe accelerated ossification in dwarf species (e.g. in teleosts: [42, 45]) and our findings in *H. zosterae* are therefore not surprising. The selective pressure for *H. reidi* to slow down bone development is unclear. According to Jones [46], disruptive selection is likely an important diversity-producing process in seahorses. In addition, seahorses of different sizes commonly occupy the same seagrass habitats and therefore there are likely multiple adaptive peaks for body size [46]. It is probably therefore that transient disruptive selection of sufficient strength occurred over adequate time periods to produce sympatric speciation in syngnathid fishes that mate assertively with respect to body size [46]. Indeed, the range of *H. reidi* entirely encompasses the range of *H. zosterae* (Western Atlantic up to Gulf of Mexico) and both occupy seagrass beds, although *H. reidi* is also found in coral reefs [7]. Overall, these results show that the evolutionary solution for *H. zosterae*, the dwarf species, was to accelerate osteogenesis. *H. zosterae* is not a developmentally truncated species but rather a proportioned dwarf, a small version of its larger ancestor. In summary, the three seahorse species studied here have tinkered with skeletal development differently in order to succeed over 20–30 million years of evolution.

### Caudal fin loss in syngnathids

In unstained adults, *H. reidi* and *H. zosterae* lack a caudal fin with fin rays whereas it is retained in *H. subelongatus*. Our data shows that larval *H. reidi* and *H. subelongatus* have remnants of the caudal fin, with an internal cartilage nodule at the end of the tail and an external fin (with fin rays). Although, no reports of a caudal fin remnant were made for *H. zosterae*[21], in Figure plate 9D in this study, it appears as if there is a knob of cartilage at the end of the tail. Interestingly, the sister clade to the seahorses, the pygmy pipehorses, comprises both pipefishes *(e.g. Filicampus)* that have a caudal fin with fin rays and the pygmy pipehorses *(e.g. Idiotropiscis)* that seem to lack a caudal fin [46]. Phylogenetically, *H. subelongatus* is more closely related to the pipefishes than either *H. reidi* or *H. zosterae* are [36] indicating that caudal fin loss is incomplete in seahorses. Earlier, Kanou & Kohno [47] had also observed the presence of a minute caudal fin composed of two fin rays hypothesized to be supported by two hypural plates (parhypural and hypural 1 + 2, and hypurals 3+) in a different *Hippocampus* species, namely *H. mohnikei* (up to 26 mm TL). In our analysis, we cannot infer homology, however it appears that the cartilage we observe in histological section corresponds to the cartilage supporting the anteriormost fin ray in the Kanou and Kohno [47] study. Teske *et al.*[36] showed that *H. mohnikei* belongs to the same clade as *H. subelongatus*, whereas *H.reidi* and *H. zosterae* are clustered in a different clade, suggesting that the presence of fin rays during earlier stages is not clade specific. This data suggests that in the *reidi-zosterae* clade the development of the endoskeletal and exoskeletal elements are ultimately de-coupled, whereas in the *subelongatus-mohnikei* clade, their development remains coupled. Interestingly, Mabee *et al.*[48] identify four modules during median fin development, one of which is an exoskeleton-endoskeleton module. A more detailed analysis of caudal fin loss in other syngnathids species including a re-examination of *H. zosterae* is needed in order to fully understand the loss of this structure over evolutionary time and how decoupling may have occurred.

## Conclusions

Based on our analyses, we can conclude that although *Hippocampus* species appear to have very similar skeletal systems, there are differences in the timing of osteogenesis across the three *Hippocampus* species studied. Heterochronic processes could explain some of the observed differences, as the skeleton in the miniaturised species reflect a proportioned dwarf (rather than a developmentally truncated dwarf). However, two similarly sized species (*H. reidi* and *H. subelongatus*) also show evolutionary changes in the onset of ossification. With respect to the grasping tail, the evolutionary loss of the caudal skeleton and fin is still apparent during development, as a highly primordial fin was observed during the early developmental stages. This study shows that these three species of *Hippocampus* seahorses have evolved different osteogenic strategies over the last 20–30 million year of seahorse evolution.
